# Gluttony

**Published:** 1876-01

**Authors:** 


The ugly Hydra, a seeming mass of jelly or sluggish flesh, whose whole body is a stomach, with fingers to fill it, stretches out its blind feelers by the hour, for any food that the water may float into its grasp; but when once filled, those fingers drop flaccid by its side, and the daintiest morsel fails to wake up their activity again. But man, a little lower than the angels, more irrational than this lowest specimen of animal life, will eat when he is already full; and, fighting against nature, will force his food against his appetite, thus sinking himself below the brutes that perish.

